# Connexin 30 Expression and Frequency of Connexin Heterogeneity in Astrocyte Gap Junction Plaques Increase with Age in the Rat Retina

**DOI:** 10.1371/journal.pone.0057038

**Published:** 2013-03-14

**Authors:** Hussein Mansour, Janet R. McColm, Louise Cole, Michael Weible, Anastasia Korlimbinis, Tailoi Chan-Ling

**Affiliations:** 1 Department of Anatomy, School of Medical Sciences and Bosch Institute, University of Sydney, New South Wales, Australia; 2 Advanced Microscopy Facility, School of Medical Sciences, Bosch Institute, University of Sydney, New South Wales, Australia; Case Western Reserve University, United States of America

## Abstract

We investigated age-associated changes in retinal astrocyte connexins (Cx) by assaying Cx numbers, plaque sizes, protein expression levels and heterogeneity of gap junctions utilizing six-marker immunohistochemistry (IHC). We compared Wistar rat retinal wholemounts in animals aged 3 (young adult), 9 (middle-aged) and 22 months (aged). We determined that retinal astrocytes have gap junctions composed of Cx26, -30, -43 and -45. Cx30 was consistently elevated at 22 months compared to younger ages both when associated with parenchymal astrocytes and vascular-associated astrocytes. Not only was the absolute number of Cx30 plaques significantly higher (P<0.05) but the size of the plaques was significantly larger at 22 months compared to younger ages (p<0.05). With age, Cx26 increased significantly initially, but returned to basal levels; whereas Cx43 expression remained low and stable with age. Evidence that astrocytes alter connexin compositions of gap junctions was demonstrated by the significant increase in the number of Cx26/Cx45 gap junctions with age. We also found gap junctions comprised of 1, 2, 3 or 4 Cx proteins suggesting that retinal astrocytes use various connexin protein combinations in their gap junctions during development and aging. These data provides new insight into the dynamic and extensive Cx network utilized by retinal astrocytes for communication within both the parenchyma and vasculature for the maintenance of normal retinal physiology with age. This characterisation of the changes in astrocytic gap junctional communication with age in the CNS is crucial to the understanding of physiological aging and age-related neurodegenerative diseases.

## Introduction

Astrocytes play an essential role in central nervous system (CNS) homeostasis via the numerous co-operative metabolic processes they establish with neurons, including neurotransmitter recycling, supply of energy metabolites and mediation of neurovascular coupling [1], [2]. Many of these astrocyte functions are mediated via gap junction (GJ) communication. Astrocyte gap junctions are formed by two hemi-channels (connexons) composed of individual homo- or hetero-hexamers of connexin (Cx) proteins. An extended field of GJs is referred to as a gap junctional plaque [for a review [3]]. To date, four connexin proteins have been found in astrocytes: Cx26 [4], Cx30 [5], Cx43 [6] and Cx45 [7] and various combinations of hexameric Cx proteins determine the types of ions and small molecules that are able to pass from the cytoplasm of one cell into the next. Thus the connexin make-up of gap-junctions has a direct influence over astrocyte syncytium communication [1], [8], [9].

Distinct astrocyte subpopulations establish different connexin expression patterns in the CNS depending on the physiological requirements of the tissue [10]. In the retina, astrocytes mostly express Cx43 but can also express Cx30 and Cx45. To date, Cx26 has not been detected in mouse or rat retina [11]. In human and rat retina, Cx43 is associated with blood vessels [12], [13] though recently, Cx30 and Cx43 have been shown to be associated only with superficial retinal blood vessels in the rat, not the deeper plexus [14], whilst Cx45 is found in cerebral smooth muscle cells [15] and rodent retinal neurons [16].

To date a number of studies have provided both immunohistochemical [4], [11], [17] and functional evidence [18]–[20] of heterotypic and homomeric coupling between astrocytes, as well as between astrocytes and oligodendrocytes [17], [18]. Zahs *et al* [11] studied the expression of Cxs in rat retinal wholemounts and concluded that since gap junction channels may be formed by both homotypic and heterotypic hemichannels, and the hemichannels may be homomeric or heteromeric, there exists a multitude of possible gap junction channels that could underlie the homotypic coupling between retinal astrocytes and the heterotypic coupling between astrocytes and Muller cells.

In order to better understand how astrocyte networks function in the aged retina, a thorough understanding of astrocyte gap junction communication as a function of aging is required. We have previously shown that astrocytes lose their ability to proliferate after 9 months, equivalent to a young adult in humans, and that the frequency of apoptosis increases significantly and persistently into old age [21]. In contrast, retinal ganglion cell numbers have been shown to remain constant in the rat retina with age [22]. Thus during aging a lower astrocyte to neuronal ratio results, placing an increased demand on the remaining astrocytes to maintain retinal homeostasis [21]. We therefore hypothesized that the remaining retinal astrocytes would alter their gap junction connexin expression, and thus communication, to compensate for the significant change in astrocyte to neuron ratio. To date, there has been very limited examination of Cx30 and 43 in the aging CNS, with only one report of decreased Cx30 number in the brain with age [23]. We undertook a thorough investigation of the aging-associated changes in astrocyte Cx expression in the rat retina with particular emphasis on Cx number, plaque size, as well as a detailed evaluation of the frequency and type of homomeric and heterotypic gap junction intercellular coupling.

Our earlier work [24], [25] has shown that the retina is an excellent model of the CNS in both normal development and aging. The absence of oligodendrocytes creates a simplified system for the study of purely astrocyte, neuronal and vascular gap junction communication. The retina has been shown to reflect CNS changes with aging, including changes in the astrocytes [21], pericytes [26], blood-retinal barrier, angiogenesis and immune response [24]. We combined the advantages offered by the whole-mount retina and developed a six marker immunohistochemistry protocol combined with confocal microscopy, spectral imaging, online emission fingerprinting and 3D image processing and analysis to visualise and quantify the total number of different Cxs and the size of gap junctional plaques. We also identified distinct heterogeneous gap junctional plaques comprised of more than one connexin protein with simultaneous visualisation of the astrocyte cytoskeleton and vasculature. Our findings lead us to suggest that retinal astrocytes display a level of plasticity in the expression of various Connexin proteins and that aging-associated changes in expression of Cxs and increased levels of heterogeneous gap junctional plaques, may play a compensatory role in GJIC and facilitate neuronal/astrocyte/vascular interactions. This modification or disruption of astrocyte GJIC during ‘physiological’ aging therefore may underlie some of the age-related changes seen in neuropathology.

## Materials and Methods

### Animals and anaesthesia

We used forty-eight Wistar rats representing the following age groups; 3 month old - young adult (equivalent to 20 years of human life), 9 month old - middle age (equivalent to 40 years of human life) and 22 month old - aged (equivalent to greater than 80 years of human life) [27], as follows: For triple maker immunostaining of each of the different connexins i.e. Cx/GFAP/GS isolectin B4, we used 4 retinas from 4 different animals at each age (total 24 animals). For Westerns, we used 6 retinas from 3 animals at each age (total 12 animals). For six-marker immunostaining (4 retinas from left eye) and area size (4 retinas from right eye), we used 8 retinas from 4 animals at each age (total 12 animals)

Animals were individually housed, given commercial rat chow, watered ad libitum and maintained as previously described [21]. For sacrifice, animals were deeply anesthetized with sodium pentobarbital (150 mg/kg) via intraperitoneal injection. All experiments were approved by the Animal Ethics Committee of the University of Sydney.

### Preparation of retinal wholemounts

Rats were perfused transcardially and retinal wholemounts prepared as previously described [21], [28]. In brief, globes were enucleated, and eyecups prepared by removing the cornea, lens and vitreous in a cold Petri dish containing 0.1 M phosphate buffer saline (PBS) pH 7.4. Retinal wholemounts were immersion-fixed with ice cold 70% v/v ethanol (−20°C) for 20 mins followed by 4% w/v paraformaldehyde (PFA) pH 7.4 for 10 mins at 4°C. Tissues were then washed thoroughly with PBS and immediately used for application of a variety of immunohistochemical procedures (see below).

### Antibodies and Markers

The following primary antibodies and markers were used in this study (see also Table 1 for more detailed information): goat polyclonal anti-human Cx26; rabbit polyclonal anti-rat Cx30; mouse monoclonal anti-human Cx43 (recognizes total Connexin 43, phosphorylated at various sites and non-phosphorylated); sheep polyclonal anti-human Cx45; chicken polyclonal anti-cow GFAP; and biotinylated Griffonia simplicifolia (*Bandeiraea*) isolectin B4 (GS isolectin B4).

**Table 1:** List of primary antibodies and markers used in this study.

Primary Antibody and Marker Name, Clone and Manufacturer	Species, Immunogen and Epitope	Antibody format, Host and Isotype	Dilution Factor	Tested and Specificity in CNS tissue	Citations
**Connexin 26** clone: N19, Santa Cruz Biotech, CA	Human Peptide-mapping to 13q12.1 at the N-terminus	Polyclonal, affinity purified, *Goat IgG*	1∶300	IHC: not tested. WB: not tested	
**Connexin 30** CZ-7200, Zymed Laboratories, San Francisco, CA	Rat Peptide-derived from C-terminus	Polyclonal, affinity purified, *Rabbit IgG*	1∶300	*Rat, Mouse & Human*. IHC: no cross-reactivity, labels astrocytes. WB: single-band	[5], [11], [17], [42]
**Connexin 43** clone: 4E6.2, Chemicon, Temecula, CA	Human Peptide- corresponding to positions 252–270 at the C-terminus	Monoclonal *Mouse IgG1*	1∶250	*Rat, Mouse & Rabbit*. IHC: no cross-reactivity, labels astrocytes. WB: single-band recognizes total Connexin 43, phosphorylated at various sites and non-phosphorylated	[11], [43], [44]
**Connexin 45** clone: GJA7, Abcam, Cambridge, MA	Human Peptide- corresponding to amino acids 354–367	Polyclonal, affinity purified *Sheep IgG*	1∶15,000	*Rat & Mouse*. IHC: no cross-reactivity, labels astrocytes (NFL), Muller & neuronal cells (IPL) WB: single-band	[11], [45]
**GFAP** clone: ab4674, Abcam,Cambridge, MA	Cow-Full length native protein	Polyclonal, affinity purified *Chicken IgY*	1: 2,000	*All Mammalian Species*. IHC: no cross-reactivity, labels astrocytes. WB: single-band	[46]–[48]
**Biotinylated Griffonia simplicifolia (Bandeiraea) isolectin B4** BS-1-B4, Sigma, St. Louis, MO	Bandeiraea Biotin conjugate	*BSI-B4* is blood group B specific & has an exclusive affinity for terminal α-D-galactosyl residues	1∶20	*Rat & Mouse*. IHC: no cross-reactivity, labels blood vessels. WB: not tested	[21], [49], [50]

IHC; Immunohistochemistry, WB; Western blot, IPL; Inner Plexiform layer, NFL; Nerve Fiber Layer.

Note - Previous work by Zahs et al, 2003 performed positive controls of Cx26 (Unique portion of cytoplamsic loop; cat_71-0500), Cx30 (Unique portion of C-terminus; cat_71-2200), -43 (aa. 252–270; cat_MAB3068), and -45 (aa. 354–367; cat_ MAB3101) antibody pre-absorption peptides against specific immunogen, and their immunostaining results demonstrate labeling of astrocytes in the rat retina (*ref.* *table 1* *&* *2* *in Zahs et al, 2003* [*11*]).

The following secondary antibodies and conjugates were used in this study: Alexa 488-conjugated anti-goat IgG, anti-mouse IgG1 (Molecular Probes, Eugene, OR, USA; 1∶2,000); Alexa 532-conjugated anti-goat IgG (Molecular Probes, Eugene OR, USA; 1∶2,000); Alexa 594-conjugated anti-sheep IgG, (Molecular Probes, Eugene, OR, USA; 1∶300); Cy3-conjugated anti-mouse IgG and anti-rabbit IgG (Jackson ImmunoResearch Laboratories, West Grove, PA; 1∶1,000 and 1∶300); Cy5-conjugated anti-chicken IgG (Jackson ImmunoResearch Laboratories, West Grove, PA; 1∶1,000); and AMCA streptavidin (Jackson ImmunoResearch Laboratories, West Grove, PA, USA; 1∶2,000).

### Antibody Controls and Optimization

To determine optimal fixation, we performed an extensive series of control experiments on rat retinal tissue, using different concentrations and combinations of fixatives (paraformaldehyde, formalin and ethanol), with or without various concentrations of Triton to permeabilize the cells, and 1% BSA/PBS to block the non-specific protein binding. Titration series and conjugate experimental controls were performed on both primary and secondary antibodies to optimise for low background noise, maximal stain sensitivity, avoidance of non-specific staining and cross-reactivity. Each secondary was species- and class-specific to each primary. We then ran a series of experiments to determine the optimal use of secondary antibody conjugates. This allowed us to optimize the fluorophores' signaling without compromising the specificity and to ensure there was no emissions spectra overlap during our 6 marker immunohistochemistry. Based on results gathered from our control experiments it was clearly important to incubate Cx26 (goat polyclonal) primary antibody and its conjugate secondary antibody first, in order to circumvent any cross-reactivity with other antibodies that were raised in goat. In addition, GS isolectin B4 needed to be applied last to avoid further non-specific staining and cross-reactivity, as well potentially increased background to noise ratio.

### Connexin immunostaining in retinal astrocytes

After our extensive series of control experiments the following optimised protocol was adopted. Fixed retinal wholemounts were permeabilised with 1% Triton-X 100 in PBS for 30 min at room temperature and non-specific binding was blocked with 1% bovine serum albumin (BSA) in PBS for 2 h before application of primary antibodies. All primary and secondary antibodies *(see above)* were diluted in 1% BSA in PBS, whilst GS isolectin B4 glycoside protein marker was dissolved in 1× Hanks' balanced salt solution (HBSS) with phenol red (Sigma, St. Louis, MO). Primary antibodies and GS isolectin B4 marker were applied overnight at 4°C, while secondary antibodies were incubated for 2 h in the dark at room temperature. Retinal wholemounts were washed three times with PBS (5 mins per wash) between each antibody application. Processed retinas were mounted in glycerol/PBS (2∶1), then mounted with cover slips and stored at −20°C prior to confocal fluorescence microscopy.

### Six marker immunofluorescence: co-visualisation of Cx26, -30, -43 and -45, GFAP and blood vessels

Immunofluorescently-stained retinal whole mounts with; Cx26 – Alexa532, Cx30 – Cy3, Cx43 – Alexa488, Cx45 – Alexa594, GFAP – Cy5, and GS isolectin B4 – AMCA, were viewed using an inverted Zeiss LSM 510 Meta confocal microscope (Carl Zeiss, Germany) equipped with a Plan-Apochromat 63×/1.40 Oil DIC M27 and excitation laser lines (405, 488, 561 and 633 nm). Z-stack images of immunolabelled astrocytes predominantly in the parenchyma and those predominantly associated with the vasculature in the retinal nerve fibre layer were acquired for analysis. In this study, as in our previous work [21], we have assigned the astrocytes that are predominantly stellate, where the process field is almost circular and radiates from the soma, and only 1–4 processes can be seen to touch a small capillary-sized blood vessel, as parenchymal. Where astrocytes processes were clearly seen to ensheath neighbouring blood vessels, we designated those as vascular-associated astrocytes. During imaging acquisition, the optimal interval (slice thickness) and pinhole size diameter were maintained at 8.2 µm and 136 µm respectively. The image frame size (512×512 pixels), scan speed (frame rate of 7) and averaging (2 scans per frame) also remained the same for all experiments. All parameters were standardised for best signal to noise ratio and adequate imaging efficacy. For six marker immunofluorescence experiments, images and spectral information were acquired using the Zeiss LSM 510 Meta confocal microscope using lambda mode configuration. In each case, a lambda stack (32 images each displaying 10 nm bandwidth from 411–754 nm) was collected; this records the spectral information for each pixel. In each case the spectral range, scan control settings [objective used (63×), pinhole size (136 µm), laser lines (405, 488, 561 and 633 nm), percentage laser transmission (7.1%), beam-splitter dichroic filter (HFT 405/488/561/633/KP 725)] were maintained the same. Reference spectra for each probe were created using regions of interest selected from the image and saved in a database (known as emission fingerprinting). Linear unmixing was performed in order to separate spectral information and the results were viewed and displayed. Linear unmixing separates the total emission signal in each pixel into weighted contributions of each dye based on the knowledge of their emission fingerprints. Six channel imaging was therefore carried out using the LSM 510 software in the online emission fingerprinting mode using the reference spectra in the database, in order to separate the different dye emissions.

All image data sets acquired with the Zeiss LSM 510 software (.lsm) were imported into Imaris software (Bitplane Inc, Saint Paul, MN) version 7.0. Utilizing the Imaris software we were able to visualise images in 3D and apply surface volume and object capability measurements of connexins based on shape, size and intensity. Snapshots of images were taken and saved as TIFF files. Imaris MeasurementPro, that allows the geometry and intensity measurement of objects to be measured, was used to quantify the number and plaque size of each connexin expressed by astrocytes. For each image, manual object segmentation was carried out using intensity thresholding for each colour channel, hence, each immunolabel as appropriate. Once objects of interest were clearly defined and distinguishable, a total number of objects per image and the size of objects were calculated. All object data (in voxels) were exported into an Excel spreadsheet.

The ImarisColoc module was used to obtain colocalisation information by identifying, visualising and quantifying colocalised regions. This latter module was used to generate a six multi-channel image of the connexins expressed by astrocytes. High-resolution images of connexin gap-junctions were processed to form three-dimensional projections via simulated fluorescence processing and orthogonal mode camera dialogue. For six-maker immunofluorescence Imaris-snapshot images, we used MATLAB complier runtime in Imaris XT function module, to set camera angle logarithmic functions and further determine measure angle, elevation and azimuth parameters. Additional imaging functions utilised included automated scale bar, object frame and clipping plane functions.

Our methodology did not permit us to distinguish between hemichannels of mixed Cx composition (i.e. heteromeric hemichannels) and heterotypic gap junction channels.

### Determination of connexin numbers and plaque sizes

Images were captured using an inverted Zeiss LSM 510 Meta confocal microscope (Carl Zeiss, Germany) equipped with Plan-Apochromat 63×/1.40 Oil DIC M27 objective and the excitation laser lines (405, 488, 561 and 633 nm). The quantitative sampling of numbers and sizes of Cx plaques on astrocytes predominantly in the parenchyma and those astrocytes predominantly associated with the vasculature in each retinal wholemount was calculated by the average mean value from eight fields of views in the central, mid peripheral and peripheral regions, as previously described [21]. These obtained values were further averaged from four retinas of different rats at each age investigated.

In the parenchymal regions, density of Cxs per mm^2^ was converted to total Cx number per retina by determining the total area of the retina. As previously described [21], the area of retina as a function of age was determined by analysis of merged photo images using Zeiss AxioVision version 4.0 acquisition software (Carl Zeiss MicroImaging, Jena, Germany) and Adobe Photoshop CS version 8.0 (Adobe Systems, San Jose, CA, USA). For the astrocytes predominantly associated with the vasculature, Cx were counted on a blood vessel branch (either artery or vein) which ran from inner to outer retina. The density of Cxs per mm^2^ of the vasculature was converted to total Cx number per retina on the vasculature by determining total blood vessel surface area using further post-capture analysis of Imaris surface function and Imaris MeasurementPro module (*see above*).

The size of each Cx plaque was measured in voxels (X, Y and Z dimensions) and pixels (2-dimensions only) following acquisition using Imaris 3D-reconstruction image analysis and processing software (Bitplane Inc, Saint Paul, MN) version 7.0. As described above, the average mean value of Cx plaque sizes were calculated from astrocytes predominantly in the parenchyma and those associated with the vasculature of the retina at each age investigated.

### Western Blots for assaying protein expression of astrocyte connexins

Each freshly dissected retina was homogenized in 50 µl cell lysis buffer (Mammalian cell lysis kit (MCL1), Sigma, St. Louis, MO; 250 mM Tri-HCl, pH 7.5, 5 mM EDTA, 750 mM NaCl, 0.5% (w/v) sodium dodecyl sulphate, 2.5% (w/v) deoxycholic acid, sodium salt, 5% (w/v) Igepal CA-630, plus protease inhibitor cocktail). Homogenates were centrifuged at 10,000 *g* for 15 min at 4°C. The supernatant was removed and the pellet was homogenised again with cell lysis buffer. The supernatants were pooled together and further centrifuged at 12,000 *g* for 10 mins at 4°C to obtain the crude membrane fraction. A micro BCA assay (Pierce Biotechnology, Inc. Rockford, IL, USA) was used to determine the protein quantification of each sample.

A 10% Tris-glycine (Bio-Rad Laboratories) pre-cast gel was used to separate the proteins, and 20 µg of protein was loaded onto each sample lane. Equal volumes of 2× sample buffer (0.5 M Tris buffer, pH 6.8, 0.5% (w/v) Bromophenol Blue, 40% (v/v) glycerol, 10% (w/v) SDS and β-mercaptoethanol) were added to each aliquot of sample. Sample mixture was heated at 95°C for 5 min to denature the protein and then loaded onto the gel. Western C protein ladder (Bio-Rad Laboratories, Gladesville, NSW, Australia) was used as the protein marker. The gels were electrophoresed for 50 min at 160 volts.

Proteins were transferred onto polyvinylidene difluoride membrane (0.45 µm) (Invitrogen, CA, USA) using Tris/glycine buffer, pH 8.3 (25 mM Tris base, 192 mM glycine, 0.1% w/v SDS, 20% v/v methanol) at 140 mA for 3 h. The membrane was blocked with non-fat powdered milk (5% w/v) for 16 h at 4°C, then incubated with the primary antibodies for a further 24 h. Dot blots were performed to optimize the titrations for primary antibodies. Primary antibody dilutions are given in Table 1. Secondary antibodies with HRP-conjugates were added for 2 h in the dark at room temperature. Horseradish-streptactin (Bio-Rad Laboratories; 1∶5000) was added with the secondary antibody to label the protein ladder. Secondary antibodies were used at the following dilutions: HRP-conjugated anti-goat IgG (R&D systems, Minneapolis, MN; 1∶1000), HRP-conjugated anti-rabbit IgG (Chemicon, Temecula, CA; 1∶15,000), HRP-conjugated anti-mouse IgG1 (R&D systems, Minneapolis, MN; 1∶1000) and HRP-conjugated anti-sheep IgG (Millipore, Billerica, MA; 1∶15,000).

The immunoreactive proteins were observed by enhanced chemiluminescence (SuperSignal® West Pico Substrate, Pierce Biotechnology, Inc. Rockford, IL, USA) in a Alpha Innotech FluorChem SP (San Leandro, CA, USA) digital imaging system with a CCD camera. Membranes were exposed for 4 mins and the images captured by the AlphaEaseFC software. The intensity of the bands were volume integrated within the linear range of the film (0.2–2 O.D.) using a Molecular Dynamics scanning densitometer. The resulting optical density measurements were used in subsequent calculations in Image J software to determine band integrated density values (IDV).

### Statistical data analysis

All graphs presented were constructed using GraphPad Prism 5.0 (La Jolla, CA, USA). Data were expressed as means ± SEM and *n* refers to the number of independent experiments per age group. One-way analysis of variance (ANOVA) were applied for quantification of connexin numbers and plaque sizes (variables) as a function of aging, with Bonferroni's correction for post-hoc multiple comparisons. Friedman's non-parametric and Dunn's multiple comparison tests were applied for analysis of protein expression of connexins. Column statistics were applied for determination of the area of the retina and vessel surface area. Statistical significance of P<0.05 were established relative to aging.

## Results

### Connexin 30 is abundantly expressed by astrocytes in aged retina

**Figure 1:**
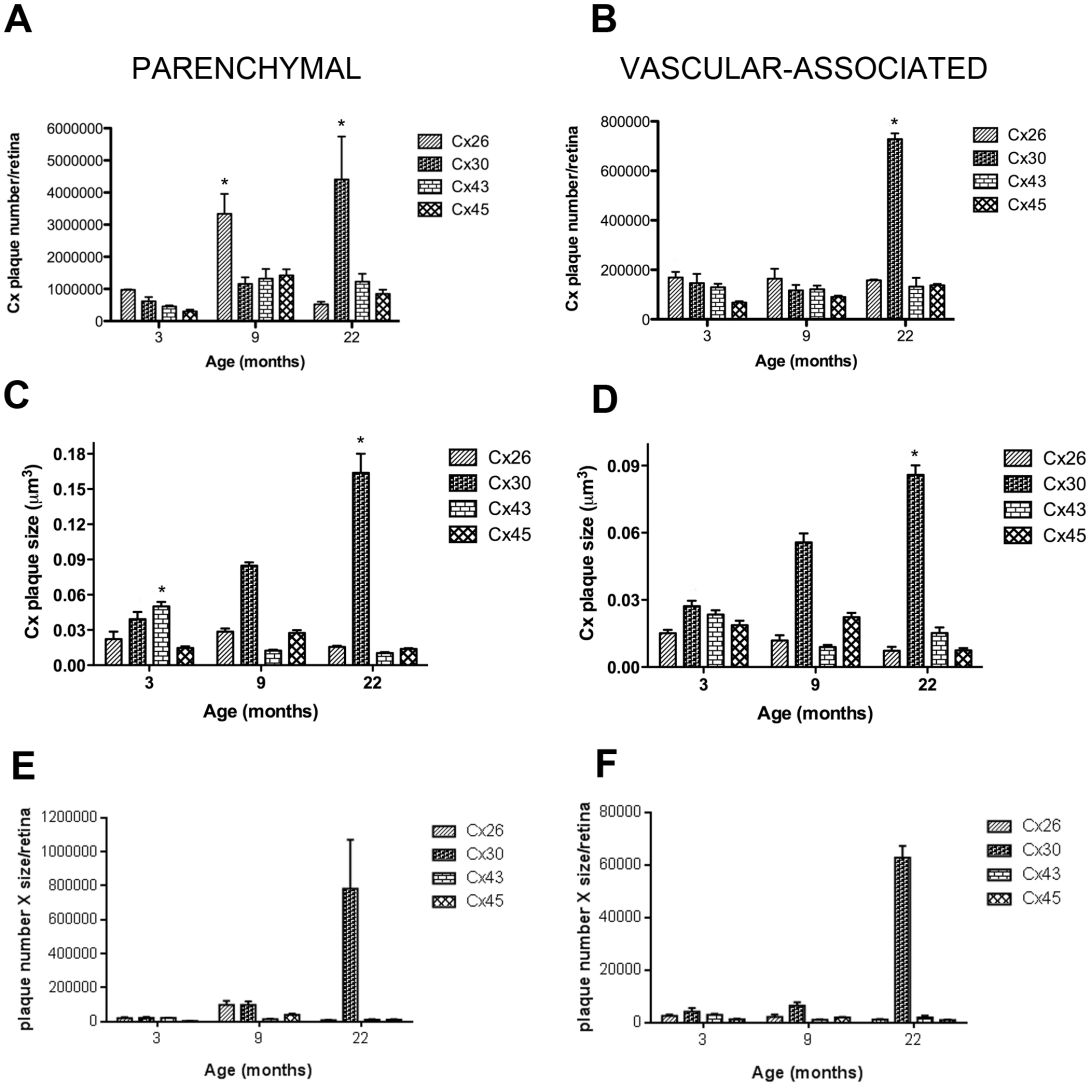
Quantification of Connexins in Astrocytes Predominantly in the Parenchyma or Predominantly Associated with the Vasculature in Rat Retina and their Differences in Aging. Changes in the number and plaque size of connexins in astrocytes predominantly in the parenchyma (A&C) and predominantly associated with the vasculature (B&D) in the retina as a function of age. At 3 months of age all connexins were detected in astrocytes predominantly in the parenchyma or predominantly associated with the vasculature. However, the plaque size of both Cx30 and Cx43 were significantly larger in the parenchyma compared to other Cx. Cx30 plaque size was larger than all other connexins at all ages associated with the vasculature. This suggest that Cx30 in young rats was the major connexin in the retina. With aging, Cx26 showed a transient increase at 9 months in the predominantly parenchymal astrocytes, but this was not sustained through 22 months. By 22 months, there was significantly more Cx30 both in the predominantly parenchymal astrocytes and astrocytes predominantly associated with the vasculature and the plaque size of Cx30 was significantly larger, compared to all other Cx. Each value represents the mean ± SEM of data from four retinas from different rats (n = 4) normalized to the total area for each retina. The mean number was also determined by counts in eight different fields of view in the central, midperipheral and peripheral regions of each retina at each age (A total of 24 fields of view per retina). One-way ANOVA with Bonferroni's post-hoc multiple comparison tests were applied for connexin plaque size and number. * denotes a statistical significance of P<0.05 for comparisons of each connexin to itself with age. E&F: the plaque number was multiplied by the size of plaque for each connexin at each time point. This confirmed our results in A–D that Cx30 was the predominant connexin associated with aging in retinal astrocytes.

Initial analyses examined aging-associated changes of Cx number and gap junctional plaque size in astrocytes averaged across three regions of the retina; central, midperipheral and peripheral By determining the total area of the retina and vessel surface area as a function of age we were able to calculate total Cx numbers and average size of gap junctional plaques in astrocytes predominantly located in the parenchyma and associated with the vasculature of the entire retina (Figure 1A & B). Since the measured area of the parenchyma was much larger than the vessel area, the counts were normalized to their respective areas as previously described [21] to allow accurate comparisons.

#### Numbers of connexin plaques

At 3 months of age all Cxs (26, 30, 43 and 45) were detected in plaques both in the astrocytes predominantly in the parenchyma (Figure 1A) and predominantly associated with the vasculature (Figure 1B). Only Cx26 in the astrocytes predominantly in the parenchyma showed a significant increase from 3 to 9 months of age (Figure 1A, p<0.05) but this was not the case for Cx26 in the astrocytes predominantly associated with the vasculature. All other Cxs plaques were not significantly different at 9 months compared to 3 months in either astrocytes predominantly in the parenchyma or predominantly associated with the vasculature. By 22 months, in astrocytes predominantly in the parenchyma or predominantly associated with the vasculature, Cx30 plaques had significantly increased compared to 3 months of age, and compared to all other Cxs at 22 months (Figure 1A and B, p<0.05).

#### Size of connexin plaques

Cx43 plaque size decreased in the parenchyma over time, with the size of the Cx43 plaque being significantly larger at 3 months compared to 9 or 22 months (Figure 1C, p<0.05). Cx43 did not show the same decrease over time when in astrocytes predominantly associated with the vasculature. In contrast, the plaque size of Cx30 increased significantly from 3 to 22 months in astrocytes predominantly in the parenchyma or predominantly associated with the vasculature (Figure 1C and D, p<0.05).

These data confirms previous results that Cx43 is present in the retina and also demonstrate that in aging the number of Cx30, and the size of plaques formed with Cx30, increases significantly.

**Figure 2:**
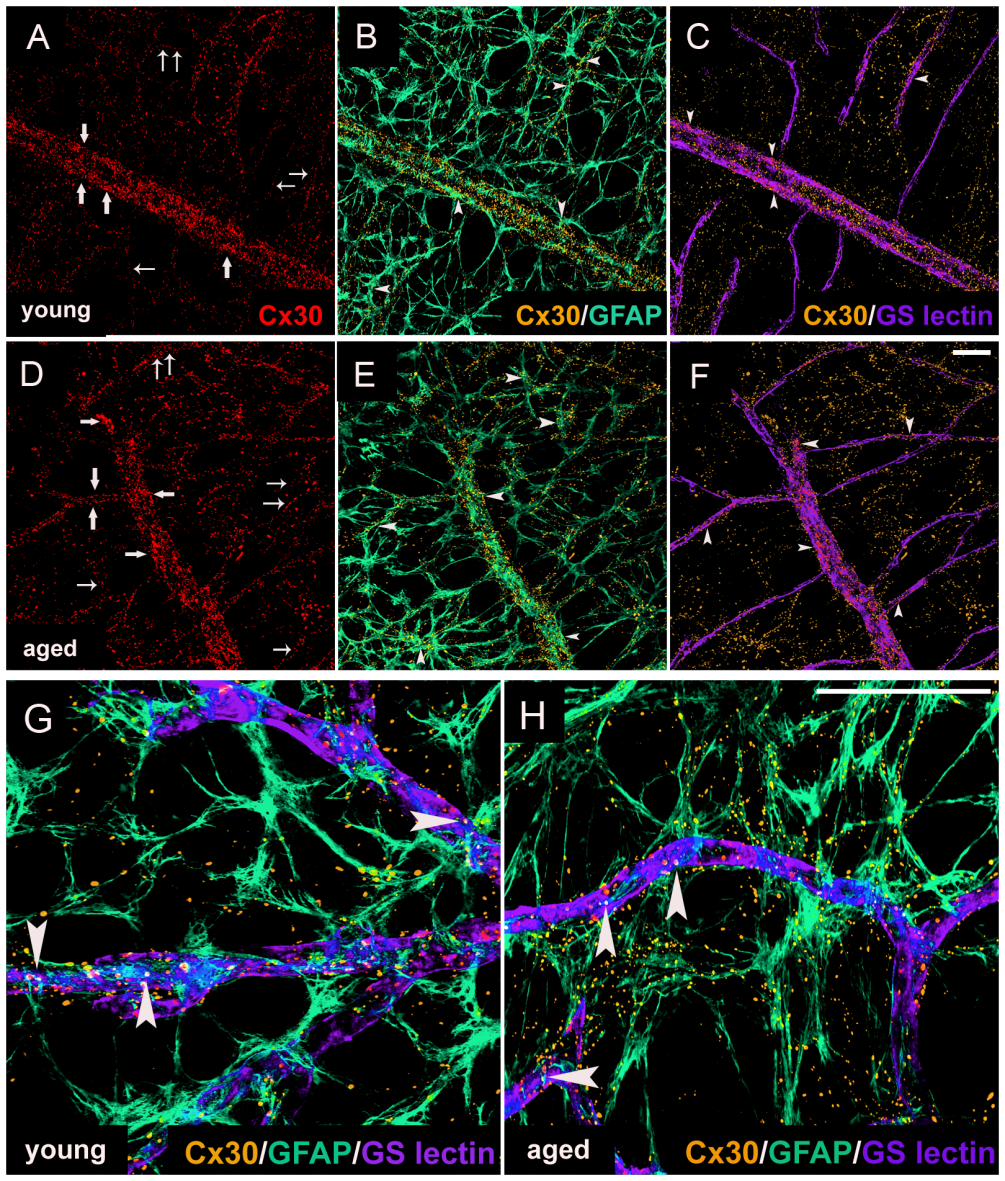
Connexin30 Expression in Astrocytes Predominantly in the Parenchyma or Predominantly Associated with the Vasculature in the Retina from Young and Old Rats. Cx30 expression in astrocytes predominantly in the parenchyma (GFAP^+^) and predominantly associated with the vasculature (GFAP^+^/GS isolectin B4^+^) in the midperipheral retina of 3-month (young adult, A–C, G) and 22-month old (aged, D–F, H) rats. At all ages examined: Cx30 (red) labelled gap junctions in astrocytes predominantly in the parenchyma (large arrows) and predominantly associated with the vasculature (small arrows). GFAP labelled astrocytes (green, B & E, G & H) and GS isolectin B4 labelled blood vessels (purple, C & F, G & H). Double immunostaining of Cx30 with GFAP or GS isolectin B4 are shown in yellow and pink respectively (arrowheads, B & E, C & F, respectively). Colocalisation of all three markers is shown in white (large arrowheads, G & H). Scale bar, 50 µm (A–H).

Cx30 was present in retinal astrocytes (Figure 2A–H), and resembled small, irregular punctate structures that were expressed throughout the retina, but especially with blood vessels (Figure 2A & D). GFAP-positive astrocytes showed classical stellate morphology typical of the fibrous astrocytes in gray matter CNS (Figure 2B, E, G & H). The increase in Cx30 numbers in aged rat retina demonstrated in Figure 1 was also apparent in the images of stained aged retina (Figure 2D–F, H) compared to young adult (Figure 2A–C, G). It would appear that more of the smaller vessels in the aged retina expressed Cx30 when compared to the young adult (Figure 2D vs A). The larger vessels appeared to have the same level of Cx30 expression at both ages assayed.

#### Protein Quantification

**Figure 3:**
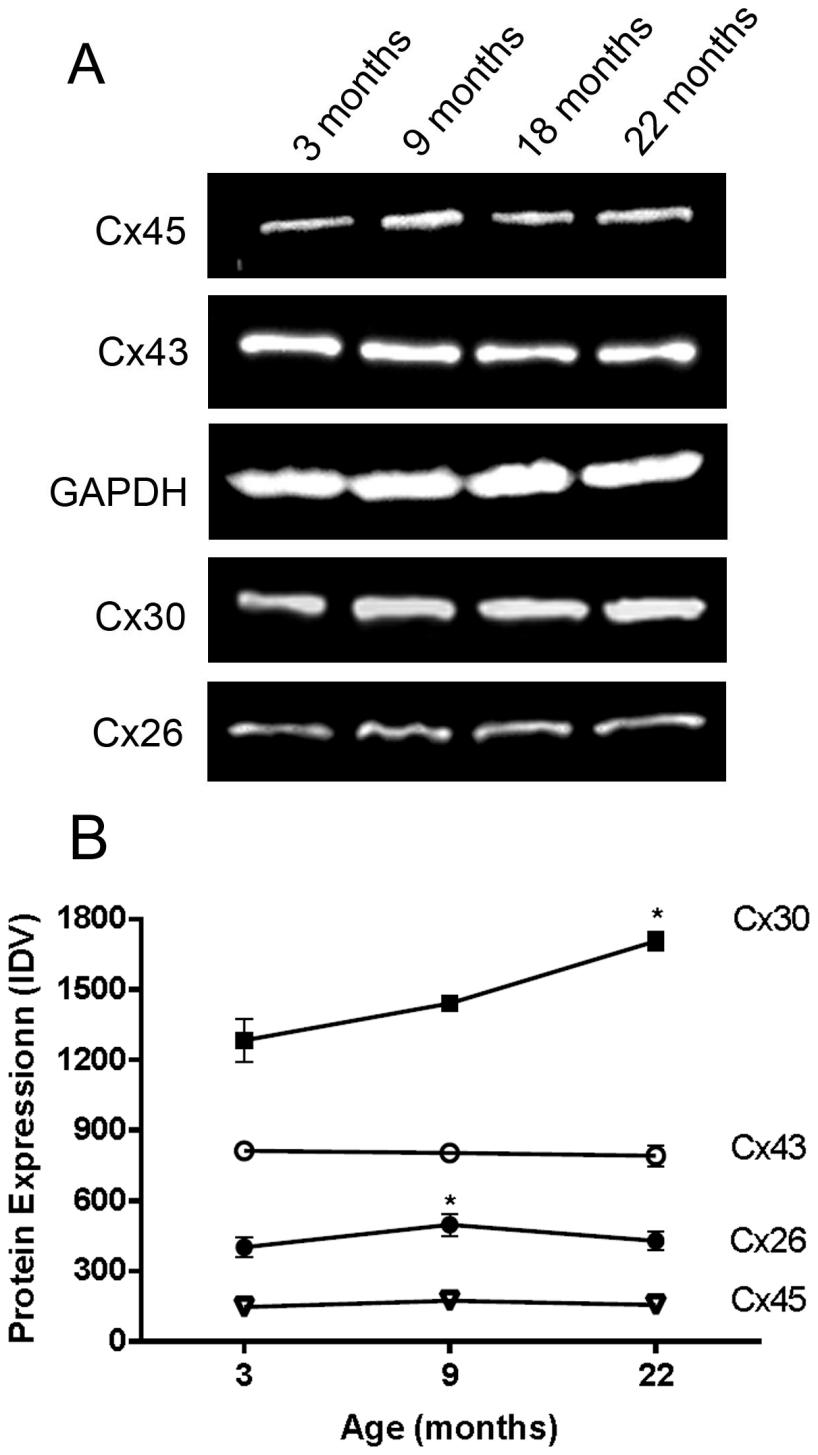
Quantification of Connexin Proteins in Young and Old Rat Retinas. Western blots using antibodies to Cx26, Cx30, Cx43 and Cx45 in rat retinal homogenates. A. Immunoblot analysis with antibodies directed to Cx26, Cx30, GAPDH, Cx43, and Cx45 were specifically detected at 25–29, 33, 37, 43, 45–47 kDa respectively. B. Band integrated density value (IDV) of each astrocyte connexin expressed during aging (except 18 months which was omitted from analysis due to insufficient numbers in this group). Each value represents the mean ± SEM of data from three retinas from different rats (n = 3). Friedman's non-parametric and Dunn's multiple comparison tests were applied. * denotes a statistical significance of P<0.05 for comparisons of each connexin to itself with age.

To further confirm our results, we assayed protein expression of all Cxs by Western blots (Figure 3A). These blots showed single bands at the molecular weight expected for each antibody applied. Thus, the antibodies were specifically detecting only the connexin they were raised against and there was no cross reactivity to other connexins. Consistent with our earlier counts, there was increased expression of Cx30 protein at 22 months of age compared to 3 month young adult (Figure 3B, p<0.05) and an increase in Cx26 protein at 9 months of age (Figure 3B, p<0.05). Expression of Cx43 and Cx45 protein showed no significant differences with aging (Figure 3B).

### Evidence of ‘connexin heterogeneity’ in gap junctional plaques in retinal astrocytes in young, middle-aged, and old rats

**Figure 4:**
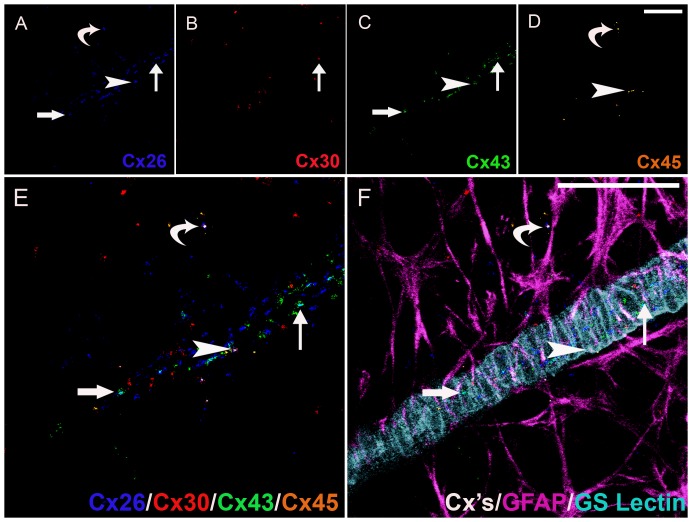
Six marker Immunohistochemistry of Connexins on Astrocytes Predominantly Associated with the Vasculature and Predominantly in the Parenchyma in the Retina of a Young Rat. Cx26 (blue, A), Cx30 (red, B), Cx43 (green, C), Cx45 (pale brown, D) were colocalised (E) on GFAP positive-astrocytes (pale purple, F). Heterogeneity in gap junction plaques are indicated as follows: Cx26^+^/Cx43^+^ (large arrows), Cx26^+^/Cx45^+^ (curved arrows), Cx26^+^/Cx30^+^/Cx43^+^ (small arrows), Cx26^+^/Cx43^+^/Cx45^+^ (arrowheads). Scale bar, 50 µm (A–F).

**Figure 5:**
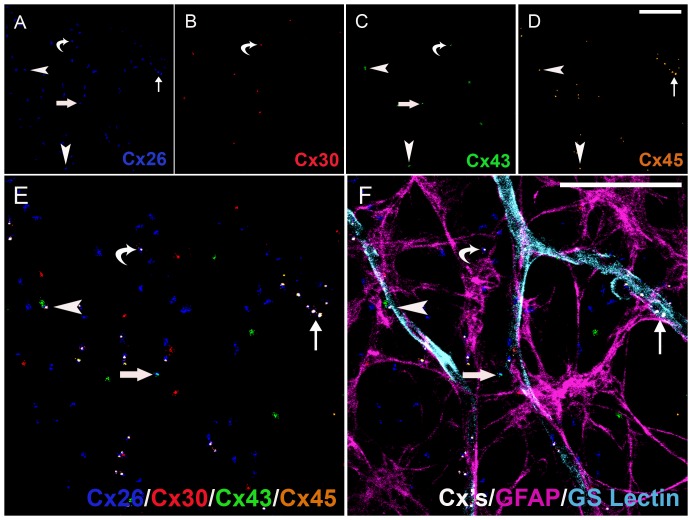
Six Marker Immunohistochemistry Connexins on Astrocytes Predominantly in the Parenchyma in the Retina of a Middle Aged Rat. Cx26 (blue, A), Cx30 (red, B), Cx43 (green, C), Cx45 (pale brown, D) were colocalised (E) on GFAP positive-astrocytes (pale purple, F). Connexin heterogeneity in gap junction plaques are indicated as follows: Cx26^+^/Cx43^+^ (large arrows), Cx26^+^/Cx45^+^ (small arrows), Cx26^+^/Cx30^+^/Cx43^+^ (curved arrows), Cx26^+^/Cx43^+^/Cx45^+^ (arrowheads). Scale bar, 50 µm (A–F).

**Figure 6:**
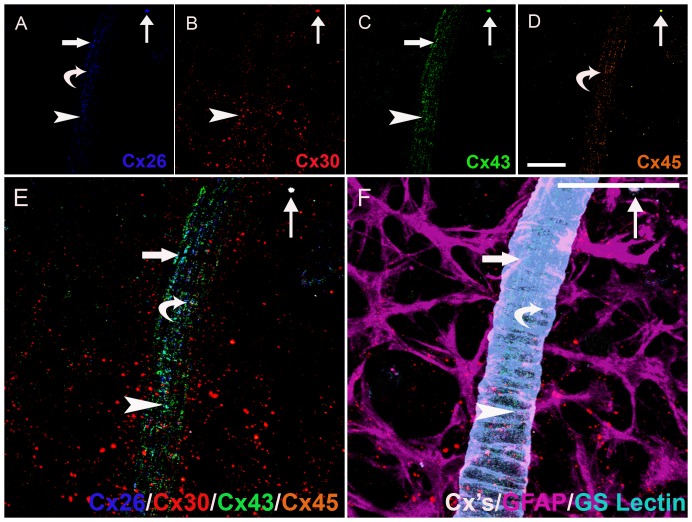
Six Marker Immunohistochemistry of Connexins on Astrocytes Predominantly in the Parenchyma in the Retina of an Aged Rat. Cx26 (blue, A), Cx30 (red, B), Cx43 (green, C), Cx45 (pale brown, D) were colocalised (E) on GFAP positive-astrocytes (pale purple, F). Connexin heterogeneity in gap junction plaques are indicated as follows: Cx26^+^/Cx43^+^ (large arrows), Cx26^+^/Cx45^+^ (small arrows), Cx26^+^/Cx30^+^/Cx43^+^ (curved arrows), Cx26^+^/Cx43^+^/Cx45^+^ (arrowheads). Scale bar, 50 µm (A–F).

Our findings of 11 distinct heterogeneous gap junctional plaques in astrocytes predominantly in the parenchyma or predominantly associated with the vasculature were qualitatively assessed utilizing advanced methods and high-throughput analysis of six marker immunohistochemistry and confocal online emission fingerprinting (Figure 4, 5, 6). Figure 4 is imaged at 3 months, Figure 5 is imaged at 9 months and Figure 6 is imaged at 22 months. In each of these figures, Cx26 (blue, A), Cx30 (red, B), Cx43 (green, C), Cx45 (pale brown, D) were colocalised (E) on GFAP positive-astrocytes (pale purple, F). Though all possible combinations of Cxs were seen (see Table 2 for a list of all possible combinations of 2, 3 and 4 Cx isoforms), only examples are shown in Figure 4, 5, 6 of: Cx26^+^/Cx43^+^ (*large arrows*), Cx26^+^/Cx45^+^ (*small arrows*), Cx26^+^/Cx30^+^/Cx43^+^ (*curved arrows*), Cx26^+^/Cx43^+^/Cx45^+^ (*arrowheads*).

**Table 2:** All possible Cx protein (26, 30, 43 and 45) combinations in retinal astrocytes.

	% change from 3 to 22 months old (Total number/retina from 3 to 22 months)	
	Astrocytes Predominantly in the Parenchyma	Astrocytes Predominantly Associated with the Vasculature
**Homomeric/Homotypic**		
Cx26/Cx26	↓ 52% (924662.2 to 437637.8)	↓ 55% (74374.1 to 32935.5)
Cx30/Cx30	**↑ 636% (592002.0 to 4355278.7)**	**↑ 562% (98788.3 to 654464.4)**
Cx43/Cx43	**↑ 176% (428717.9 to 1185838.8)**	↓ 2% (60393.7 to 58757.8)
Cx45/Cx45	**↑ 183% (267653.5 to 758149.4)**	↑ 35% (10821.6 to 14634.7)
**Heteromeric/Heterotypic**		
**Two Cx**		
Cx26/Cx30	↓ 20% (11168.9 to 8912.0)	↓ 45% (22540.9 to 12288.1)
Cx26/Cx43	↓ 25% (9988.3 to 7409.4)	↓ 55% (28875.2 to 12879.0)
Cx26/Cx45	**↑ 159% (16387.4 to 42487.8)**	**↑ 171 (21548.9 to 58597.5)**
Cx30/Cx43	↑ 76% (3471.1 to 6114.1)	↑ 71% (7105.6 to 10188.1)
Cx30/Cx45	**↑ 422% (2526.5 to 13212.6)**	**↑ 146% (5153.2 to 12694.2)**
Cx43/Cx45	↑ 6.3% (6776.9 to 7202.2)	↓ 6.2% (11896.1 to 11155.5)
**Three Cx**		
Cx26/Cx30/Cx43	↑ 72% (2550.2 to 4391.5)	↑ 72% (5444.8 to 9419.1)
Cx26/Cx30/Cx45	**↑ 421% (1700.1 to 8860.2)**	**↑ 282% (2767.3 to 10576.9)**
Cx26/Cx43/Cx45	↑ 70% (4746.2 to 8083.0)	↑ 7.5% (10777.8 to 11587.3)
Cx30/Cx43/Cx45	**↑ 232% (1558.4 to 5181.4)**	**↑ 314% (2209.7 to 9149.3)**
**Four Cx**		
Cx26/Cx30/Cx43/Cx45	**↑ 248% (1487.6 to 5181.4)**	**↑ 258% (2637.3 to 9452.2)**

These different Cx protein combinations were quantified as % change with age (3 vs 22 months comparison). Statistically significant increases from 3 to 22 months were calculated in astrocytes predominantly in the parenchyma or predominantly associated with the vasculature and are shown in bold (see also Figure 8).

**Figure 7:**
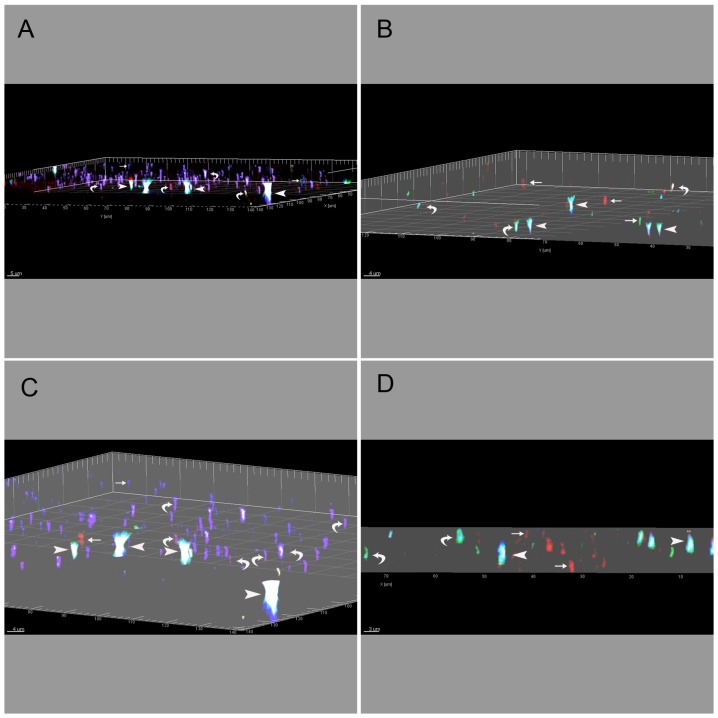
Imaris Snapshot Images through the Entire Depth of the Retinal Nerve Fibre Layer. Imaris snapshot images of confocal z-stack projections that visualise the entire depth of the retinal fibre layer where both astrocytes and blood vessels are situated. The overlapping immunohistochemistry represents connexin heterogeneity in gap junction plaques expressed by astrocytes and captured using high resolution three-dimensional projection, simulated fluorescence processing and orthogonal mode camera dialogue. The GFAP and GS isolectin B4 stain were omitted from these images to better illustrate the fluorescence of the connexins. A&C were taken in the central retinal region and B&D from a peripheral retinal region. A&C and B&D are a different angle of the same 3D analysis to show different connexin combinations as indicated by the arrows. Immunostaining of astrocyte connexins represents Cx26 (blue), Cx30 (red), Cx43 (green) and Cx45 (brown). Different colocalisation patterns of connexin proteins in diverse astrocyte connexin hemichannels were qualitatively analysed (1Cx protein, small arrows, 2Cx and 3Cx proteins, curved arrows, and 4Cx proteins, large arrowheads). All images are highly zoomed and rendered confocal z-slice projections. For qualitative purposes, displayed images were also constructed using automated scale bar, object frame (grid, tickmarks and box) and XYZ clipping plane (i.e. crops object below the plane). The set camera angle logarithmic functions were also applied to measure angle, elevation and azimuth parameters for each of the images represented. This was as follows: *angle* = −5.6521 (A), 4.4572 (B), −6.3247 (C), −0.0729 (D), *elevation* = 91.8453 (A), 88.6771 (B), 93.7761 (C), 90.0196 (D), *azimuth* = 119.9393 (A), −65.222 (B), 127.6513 (C), 93.3840 (D).

**Figure 8:**
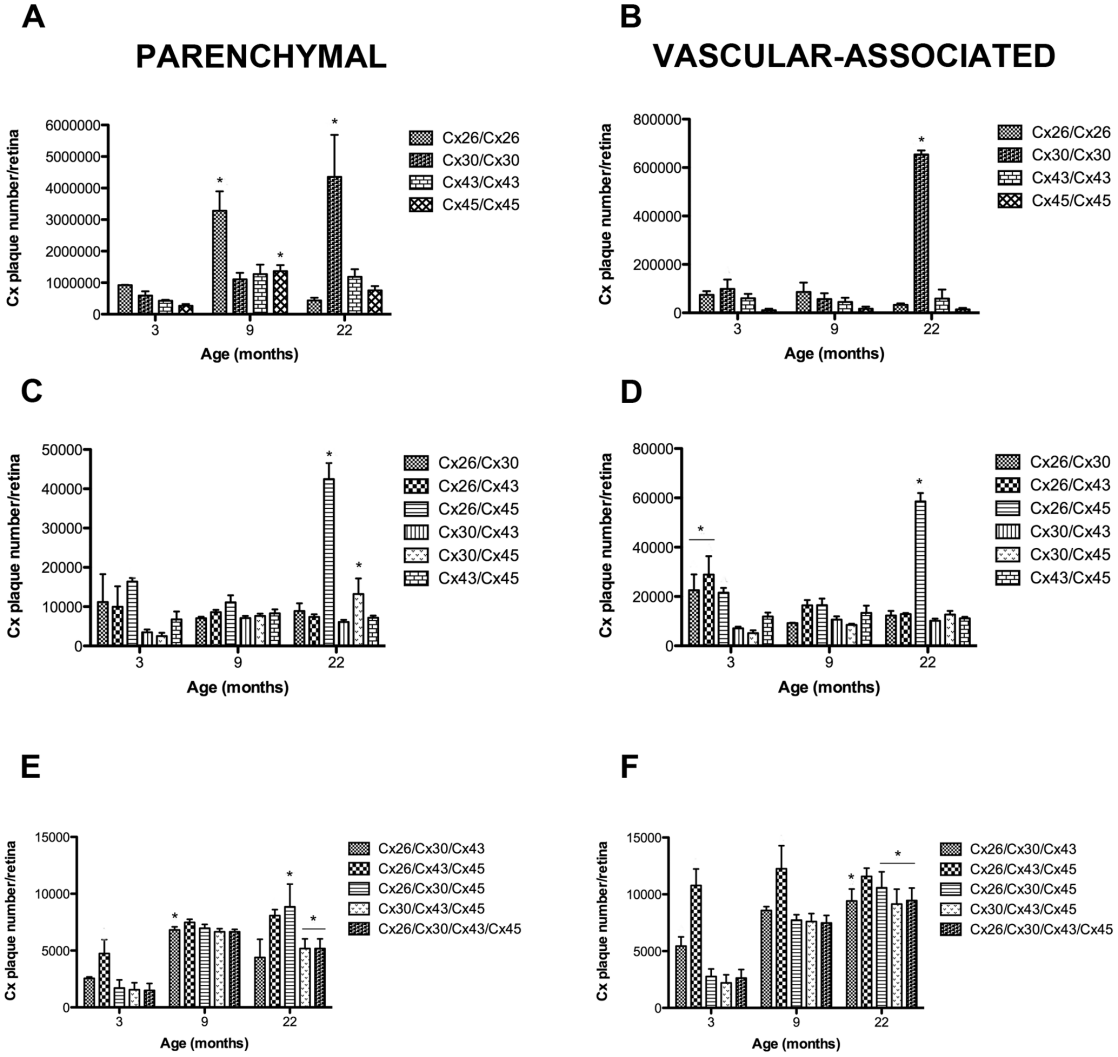
Quantification of the Connexin Heterogeneity in Gap Junction Plaques During Aging in the Rat Retina. Changes in the number and type of connexins in gap junction plaques in astrocytes predominantly in the parenchyma (A, C &E) and predominantly associated with the vasculature (B, D & F) in the retina as a function of age. In all graphs, each value represents the mean ± SEM of data from four retinas from different rats (n = 4). The mean number was also determined by counts in eight different fields of view in the central, midperipheral and peripheral regions of each retina at each age (a total of 24 fields of view per retina) and normalized to the total area for either the parenchyma or vasculature. In each graph, x-axis represents age in months, and y-axis represents number per retina (A–F). One-way ANOVA with Bonferroni's post-hoc multiple comparison tests were applied. * denotes a statistical significance of P<0.05 for comparisons of each connexin to itself with age. The horizontal line indicates all these Cx heterogeneous GJs at the particular age time point were significantly different compared to 3 months of age.

Imaris image processing allowed visualisation of complete retinal cytoarchitecture in three-dimensions and high resolution protein interaction. Simulated fluorescence and orthogonal dialog camera projection were also applied to further ascertain the distinct heterogenous gap junctional plaques in retinal astrocytes (Figure 7). Figure 7 illustrates image snapshots via high-throughput processing with respect to zoom, maximal intensity, coordinate position and shading acquisition. This is the first demonstration of co-visualisation of four Cx protein combinations (Cx26/Cx30/Cx43/Cx45) in astrocytic GJ's. We were able to identify all six of the possible combinations comprising two Cx's; all four of the combinations comprising three Cx's and the only combination comprising all four Cx's (see Table 2 for all possible Cx isoform combinations; Figure 8) as well as the four homomeric-homotypic combinations: Cx26/Cx26, Cx30/Cx30, Cx43/Cx43and Cx45/Cx45.

### Homomeric-Homotypic Combinations are Most Abundant

Figure 8A and B demonstrate that the homomeric-homotypic combinations of both predominantly parenchymal and predominantly vascular-associated astrocytes are the most abundant in the retina. Not surprisingly, Cx26/Cx26 in the predominantly parenchymal astrocytes mirrors the transient rise at 9 months of age seen with the Cx26 protein alone (Figure 1A). We also demonstrate that Cx30/Cx30 is significantly elevated at 22 months compared to all other Cx homomeric-homotypic combinations, again confirming earlier results of Cx30 protein alone. In the predominantly vascular-associated astrocytes there is a slight elevation of Cx30/Cx30 over the other Cx combinations at 3 months, but this increase is much more pronounced, and significant, with age (Figure 8B). These results again confirm that Cx30 is a major retinal connexin protein.

### Increased numbers of heterogeneous gap junction plaques in retinal astrocytes with aging

Quantitation of quadruple-label connexin immunoreactivity identified five combinations of heterogeneous connexin gap junctional plaques—that may either form heteromeric, mixed-homotypic and heterotypic interactions—that showed statistically significant increases with age; Cx26/Cx45, Cx30/Cx45, Cx26/Cx30/Cx43/Cx45, Cx30/Cx43/Cx45 and Cx26/Cx30/Cx45 (Figure 8).

During aging, there was a significant 2-fold increase in the number of Cx26/Cx45 GJs in astrocytes predominantly in the parenchyma or predominantly associated with the vasculature at 22 months of age (Figure 8C & D, p<0.05), even though both of these individual Cxs were expressed at lower levels at this age (Figure 1A&B, and Figure 3B).

Expression of Cx30/Cx45 in astrocytes predominantly in the parenchyma was also found to significantly rise 3-fold with age (Figure 8C, p<0.05). Other double Cx protein combinations including Cx26/Cx30, Cx26/C43, Cx30/Cx43 and Cx43/Cx45 also were not significantly different in number in astrocytes predominantly in the parenchyma or predominantly associated with the vasculature at 22 months aged compared to 3 months young adult.

Expression of Cx26/Cx43/Cx45 was highest compared to other triple and quadruple Cx protein combinations in astrocytes predominantly in the parenchyma or predominantly associated with the vasculature at 3 months (Figure 8E & F, p<0.05) but this was only true for astrocytes predominantly associated with the vasculature at 9 months (Figure 8F, p<0.05).

Cx26/Cx30/Cx43 in astrocytes predominantly associated with the vasculature was upregulated at 3 months and was significantly different at 22 months (Figure 8F, p<0.05). Cx26/Cx30/Cx45, Cx30/Cx43/Cx45 and Cx26/Cx30/Cx43/Cx45, in astrocytes predominantly in the parenchyma or predominantly associated with the vasculature, also demonstrated a significant 2-fold upregulation at 22 months compared to 3 months of age (Figure 8E & F, p<0.05).

## Discussion

### Connexin 30 is the principal astrocytic gap junction protein expressed during retinal aging; Cx43 remains relatively unchanged

Cx30 is an important astrocyte connexin in the CNS and is highly expressed in both brain and retinal astrocytes [5], [11]. There are marked regional differences in the expression of Cx30 however, with gray matter expressing more Cx30 relative to white matter [5]. In our studies of the rat retina, which is consistent with gray matter CNS, Cx30 was expressed predominantly in astrocytes and was found localised as either regular or irregular, dense puncta primarily present on the blood vessels. These results confirm previous studies in developing and adult rat retina [11], [17], and rat brain [5], that Cx30 is expressed where astrocytes interact with blood vessels. The late onset of Cx30 expression during development [5], and in cell culture studies [5], [17], suggest that Cx30 is associated with communication between relatively mature astrocytes. Our immunohistochemistry results confirm this observation and we show for the first time that in aging retina Cx30 expression increases, measured both by counting numbers of Cx30 and plaque sizes, and the product of both. We also confirmed an increase in Cx30 by measuring total protein levels.

As has been previously reported [12], [13], we confirm that Cx43 was readily detectable in rat retinal tissues at all ages. However, there was no aging associated increase as demonstrated by Cx30.

Our results differ to those of Cotrina *et al.* [23], who demonstrated that in mouse brain astrocytes, both Cx30 and Cx43 expression peaked at 7 months; Cx43 plaques remained elevated at 21 months while the number of Cx30 plaques dropped by half; and Cx30 and Cx43 protein expression remained constant during aging. Differences to our results could be due to regional and species differences since Cotrina et al., studied sections of mouse hippocampus, whilst we studied rat retinal wholemounts and differences in methodological approaches. Cotrina *et al.* utilised single immunohistochemistry on adjacent serial sections and we utilized six marker immunohistochemistry concurrent with advanced post-capture image analysis. Cotrina *et al.*'s method seriously limits the accuracy of GFAP and Cx co-localisation. Cotrina et al., also measured Cx levels in whole brain extracts rather than in hippocampus, which is where they measured their Cx expression. A further concern is their use of the Fluorescence Recovery After Photobleaching (FRAP) technique to evaluate functional gap junction coupling. CDCFDA, the dye used in the FRAP technique, is a non-fluorescent molecule that diffuses into cells and is hydrolyzed by intracellular non-specific esterases, yielding a membrane-impermeant fluorescent probe. The probe accumulates exclusively in cells with intact cell membranes. However, this probe labels all intact cells in the hippocampal slices and not exclusively astrocytes. Therefore Cotrina *et al.* can only claim that coupling between *all* cells present in the hippocampus, including neurons and oligodendrocytes, remains high not that astrocyte coupling remains high.

### Heteromeric, Mixed-Homotypic and Heterotypic Interactions in Retina

While the most abundant combinations in the retina were homomeric-homotypic, we readily detected all possible combinations of connexin heterogeneity in gap junction plaques that may either form heteromeric, mixed-homotypic and heterotypic interactions in astrocytes in the rat retina. The Cx26/Cx45 combination was found to be abundantly expressed and significantly upregulated with age. A further four combinations of connexin proteins: Cx30/Cx45, Cx26/Cx30/Cx45, Cx30/Cx43/Cx45 and Cx26/Cx30/Cx43/Cx45 were also found to significantly increase with aging. This is the first demonstration of aging-associated changes in heterogeneous gap junctional plaques in astrocytes in the CNS.

Altevogt and Paul [29] showed that astrocytes form two distinct classes of gap junctions with each other: one containing Cx26 and the second containing Cx30 and Cx43. Another study also demonstrated that astrocyte Cx26 co-localises with Cx30 and Cx43 in the CNS [4]. Our observations, in combination with these previous studies, support the suggestion that Cx30 and Cx43 interact to form heteromeric connexons, and that these second class of astrocyte gap junction consists primarily of heteromeric, heterotypic intercellular channels. Our observations concur with previous findings that there is an unexpected complexity for gap junctions between astrocytes. The occurrence of these naturally occurring heteromeric, heterotypic intercellular channels could subserve unique function. However, significant additional functional studies are required to support this hypothesis.

### Functional Significance of Heterotypic Gap Junctions

Bevans *et al.* 1998 [30] demonstrated that heterotypic gap junctions could discriminate which second messenger molecules could pass through them and this was partially due to the pore diameter. There is also evidence that hetero-mixing of multiple Cxs in astrocytes may contribute to directionality of substances which pass through GJ's [18]. Astrocytes have an important role in potassium buffering during neuronal activity and it has been suggested GJs in astrocytes provide an intracellular pathway for the removal of potassium into available sinks such as capillaries. Evidence for this is that astrocyte Cx30 co-localizes with large inwardly rectifying potassium channels in adult brain [5]. Whilst our confocal imaging suggests that various patterns of heterotypic and heteromeric appearance of glial connexins exist in the rat retina, further functional studies would provide additional support for this interpretation.

### Connexin plaque size and conductance are related

We found that plaques that consisted of Cx30 were increased in size with aging. Whilst earlier reports [31], [32] suggested that only 10% of gap junction channels are open, Nakajima's conductance measurements were compared with plaque size assuming 100 pS single channel conductance. We now know the unitary conductance of Cx36 (the mammalian orthologue of the fish Cx35) is about 15 pS, thus Nakajima's conclusion that only a small percent of connexons in gap junction plaques represent open intercellular channels is incorrect. Further, more recent studies by Bukauskas [33] also found similar results, again comparing plaque size (using GFP tagged connexins) with conductance. Thus, there is currently no doubt that plaque size and conductance are related and that a higher immunosignal between cells can be interpreted as more communication between cells, though the exact relationship between plaque size and conductance remain to be determined.

### The Dynamic nature of connexin expression by astrocytes: Friend or foe in Aging and Disease?

Taken together with previous findings our results lead us to suggest a mechanism whereby astrocyte-neuronal-vascular interactions during aging results in an altered pattern of Cx expression as reported in this study. We have recently demonstrated that the ratio of astrocytes to neurons decreases significantly with age, [21] and we know that neurons affect the expression of Cx in astrocytes in the CNS [34], particularly Cx30 and 43 [35].Thus we suggest that a feedback mechanism exists whereby neurons determine the changes in astrocytic Cx and gap junctional plaques expression during aging as the astrocyte-neuronal ratio decreases.

Further, our earlier work has shown major structural changes in the retinal vasculature with aging, including a breakdown of the blood-retinal barrier, dilatation, tortuosity and active angiogenesis [24]. We propose that these aging-associated vascular changes underlie the increase in heterogeneity of Cxs observed in astrocyte gap junctions in the aged rat retina, since all five of the Cx proteins combinations (Cx26/Cx45, Cx30/Cx45, Cx26/Cx30/Cx45, Cx30/Cx43/Cx45 and Cx26/Cx30/Cx43/Cx45) included Cx45 which has been shown to be essential for angiogenesis and other vascular functions [36]. Based on the above, our data leads us to conclude that astrocytes have the ability to dynamically regulate the expression of Cx to best subserve their functional roles in the aging CNS. Further work will be necessary to provide compelling evidence for this interpretation of our findings.

Changes in Cx expression in astrocytes have been reported in disease. Studies have shown that increased astrocyte Cx43 is associated with glaucoma [37] and Huntington disease [38]. It has also been demonstrated that Cx43 is altered in experimental diabetic retinopathy in rat retinal astrocytes [39] and mutations in Cx43 lead to small eyes and often blindness in the syndrome oculodentodigital dysplasia (ODDD) [40]. Ball *et al.* 2011 [41] also showed Cx30 to be reduced in diabetic rat brain astrocytes, however this was dependent on the region of the rat brain studied. As we have now demonstrated an increase in astrocyte Cx30 with aging, and several astrocyte Cx gap junction couplings all expressing Cx45, we suggest that these changes in astrocyte function during aging may underlie the pathology of various neurodegenerative conditions associated with aging including glaucoma.
